# Evaluation of Environmental Risk Due to Metro System Construction in Jinan, China

**DOI:** 10.3390/ijerph14101114

**Published:** 2017-09-25

**Authors:** Guo-Fu Wang, Hai-Min Lyu, Jack Shuilong Shen, Lin-Hai Lu, Gang Li, Arul Arulrajah

**Affiliations:** 1 Jinan Rail Transit Group Co., Ltd., Jinan 250101, China; metro_jinan@126.com; 2 Collaborative Innovation Center for Advanced Ship and Deep-Sea Exploration (CISSE), Department of Civil Engineering, Shanghai Jiao Tong University, Shanghai 200240, China; 13122180763@163.com (L.-H.L.); gang.li@live.cn (G.L.); 3 Department of Civil and Construction Engineering, Swinburne University of Technology, Hawthorn, VIC 3122, Australia; aarulrajah@swin.edu.au

**Keywords:** Jinan City, groundwater system, AHP, GIS, metro line construction

## Abstract

Jinan is a famous spring city in China. Construction of underground metro system may block groundwater seepage, inducing the depletion risk of springs. This paper presents an assessment of the risk due to metro line construction to groundwater in Jinan City using Analytic Hierarchy Process (AHP) and Geographic International System (GIS). Based on the characteristics of hydrogeology and engineering geology, the assessment model is established from the perspectives of surface index and underground index. The assessment results show that the high and very high risk levels of surface index exceed 98% in the north region; and high and very high risk levels of underground index exceed 56% in urban center and southern region. The assessment result also shows that about 14% of the urban area belongs to very high risk level; regions of high risk are 20% in urban area, 9% in Changqing County and 43% in Pingyin County. In the high risk region, metro lines R1 to R3, which are under construction, and metro lines L1 to L5, which are planned, have very high and high risk. Therefore, risk control measures are proposed to protect the groundwater seepage path to spring.

## 1. Introduction

Jinan City is the capital city of Shandong Province, China. Spring basin is a groundwater catchment, where groundwater resources gather and form into springs. Jinan, a historic city of China, is famous as the “Spring City” because of its numerous spring groups [1]. Jinan City is also named the Jinan karstic spring basin for its abundant groundwater, cropping out to the surface into fountains. Jinan karstic spring basin is a closed groundwater system, where the recharge, runoff, and discharge are completely independent [2,3,4]. The spring basin includes the main spring group areas and the adjacent areas within the drainage separated by Yellow River. Besides spring groups, the hydrological characteristics of aquifer is also rich in groundwater. The spring groups and rich aquifer groundwater together compose the special groundwater system in Jinan City [5].

With the population boom, rapid urbanization has been conducted in many Chinese cities recently. Large numbers of infrastructures, e.g., high railway tunnels, underground stores, and underground parking garages, have been constructed [6,7,8,9,10,11]. In recent years, many metro lines have been constructed or are under construction to alleviate traffic tension [12,13,14]. However, at the same time, many engineering accidents associated with underground excavation frequently occur [15,16,17]. To mitigate the accidents induced by underground constructions, Shen et al. [14,18,19,20] proposed a series of ground improvement technologies in the region of soft deposit (Shen et al.) [14,21]. Wu et al. [9,22,23] also proposed a new method to predict geo-hazards in the process of tunnel construction. 

Moreover, since the construction of underground infrastructures changes the natural geological environment, impact on urban geological environment, especially on the groundwater seepage condition, will also occur. Underground constructions inevitably block groundwater seepage environment [24,25,26,27,28,29]. In recent years, many underground spaces are planned in Jinan [5,30]. At present, three metro lines are under construction and five metro lines are planned. By the end of 2023, the eight metro lines with the total length of 245 km will be constructed in Jinan [31,32]. During the process of underground construction, withdrawal of groundwater has caused environmental problems in Jinan, such as karst collapse hazards, land subsidence, and non-spraying of spring [2,3,33,34,35]. Therefore, the interactions among geological and hydrogeological environment, springs, and metro lines development have to be concerned. To mitigate the negative influence of environment, this paper employs the AHP method and GIS technology to evaluate the risk of metro line construction to groundwater system in Jinan. 

The objectives of this paper are: (i) to evaluate the risk level from the perspectives of surface index and underground index based on the characteristics of topography, hydrogeology and geology of Jinan; (ii) to evaluate the regional risk of metro lines construction to groundwater system; and (iii) to discuss the risk level of metro lines, which are under construction and planned based on the assessed regional risk level.

## 2. Study Area and Data Sources

### 2.1. Study Area

**Figure 1:**
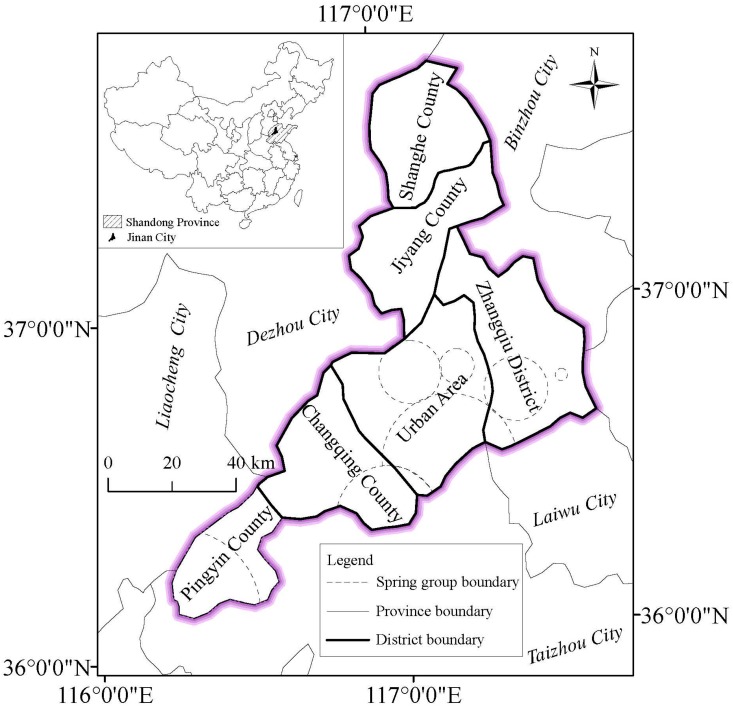
Map of administrative region and distribution of spring group in Jinan.

Jinan, a historic city of China, is famous due to the Jinan karstic spring basin, and its abundant groundwater cropping out to the surface into fountains. Jinan karstic spring basin is a closed groundwater system, where the recharge, runoff and discharge of groundwater are completely independent. Jinan is the capital city of Shandong Province, China, lying within latitudes 36° N to 37° N and longitudes 116° E to 117° E. Figure 1 depicts the map of administrative region and distribution of spring groups in Jinan. The area of Jinan is about 8177 km^2^, including Pingyin County, Changqing County, Zhangqiu District, Jiyang County, Shanghe County, and urban area. In recent years, Jinan City is planning to construct metro lines from urban area to suburban areas. At present, metro lines R1, R2 and R3 are under construction and metro lines L1, L2, L3, L4 and L5 are planned in urban area. As shown in Figure 1, Jinan City is abundant with spring groups, particularly in urban area. The construction of metro system is bound to cause damages to spring groups, which may induce serious environmental problem. To protect the spring and groundwater systems of Jinan, it is significant to evaluate the risk and impact of metro line construction on the specific karstic spring distribution and groundwater system [10].

### 2.2. Data Sources 

#### 2.2.1. Topography

**Figure 2:**
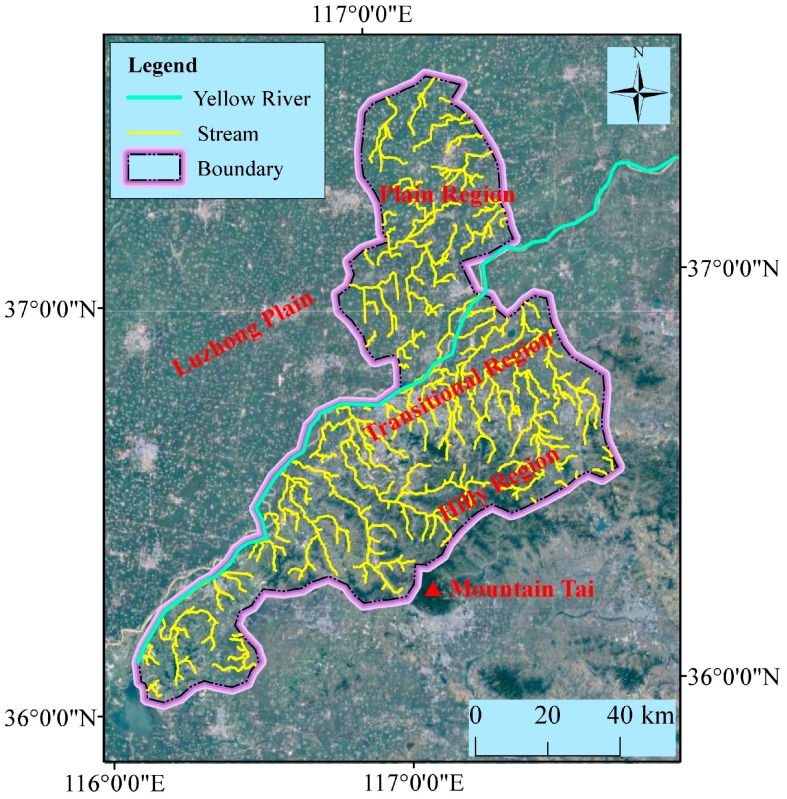
Topography surrounding Jinan.

**Figure 3:**
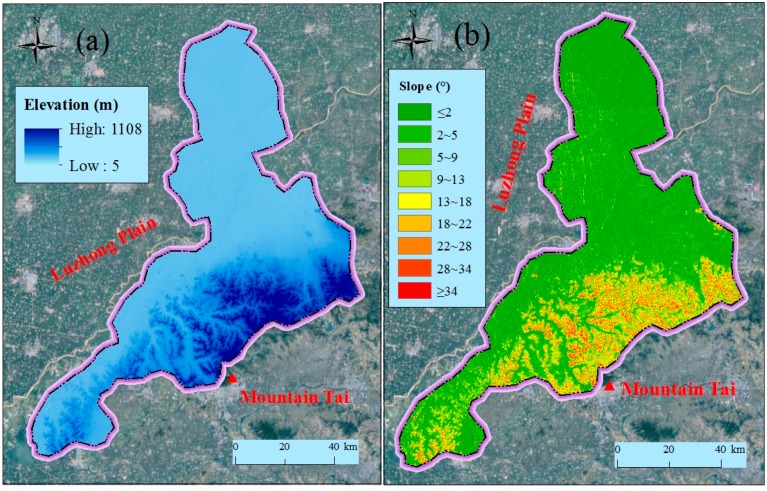
Spatial distribution of topographical elevation and slope in Jinan: (**a**) elevation; and (**b**) slope.

Figure 2 shows the topography of Jinan administrative region. Jinan administrative region is located in the south of Luzhong plain and neighbors Mountain Tai in the south, and Yellow River passes through the northern part of Jinan (see Figure 2). The terrain region of Jinan is therefore characterized by low elevation at the north side and high elevation at the south side. As shown in Figure 2, the terrain area of Jinan includes three types: plain region, hilly region, and transition region. The plain region is the alluvial plain of the Yellow River with the altitude from 5 m to 8 m; the hilly region is at the north boundary of Mount Tai with an elevation of 250 m to 1108 m; and the transition region is the piedmont alluvial plain between the plain region and hilly region. Moreover, Jinan has intensive distribution of river system. Figure 3 shows the topographical elevation and slope in Jinan. As shown in Figure 3a, the elevation is lower in the north than in the south. Figure 3b shows the slope of Jinan. As shown in Figure 3b, the slope is flatter in the north than in the south. These topographical characteristics of Jinan cause the lower regions to readily suffer from geohazards, e.g., debris flows, landslides, and flooding.

#### 2.2.2. Hydrogeology

**Figure 4:**
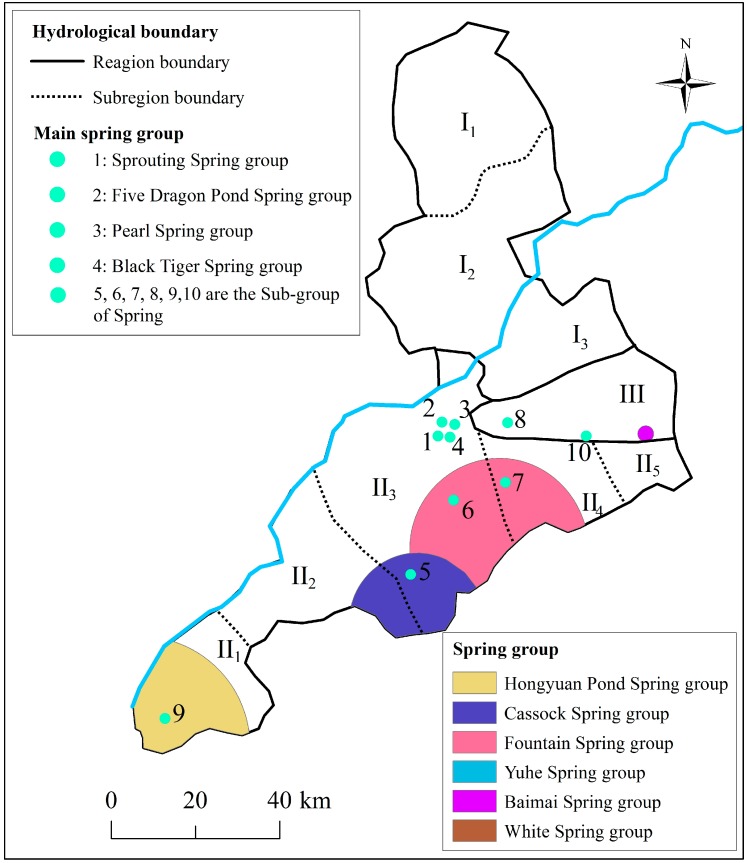
Spatial distribution of the karstic spring group in Jinan City.

**Figure 5:**
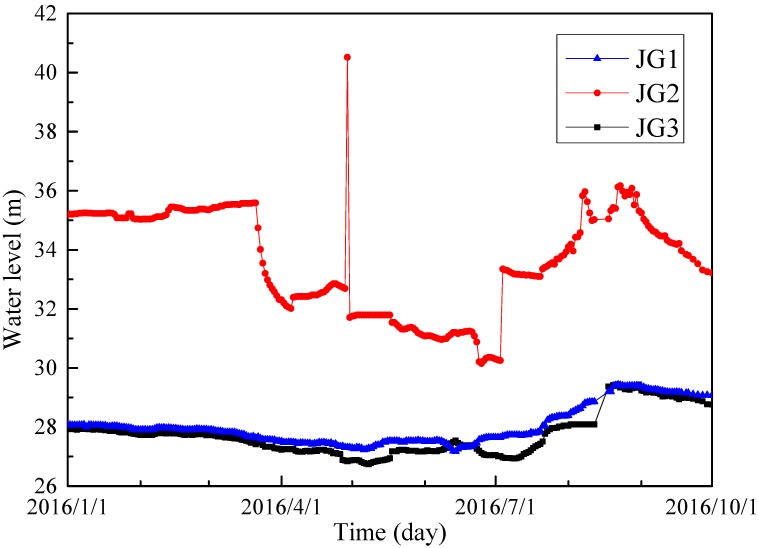
Water level of Sprouting Spring monitored in 2016.

Metro construction can disturb the balance of groundwater system. It is essential to assess the risk and impacts of groundwater system due to metro construction, especially in the “Spring City”. Figure 4 shows the spatial distribution of the karstic spring within the urban center of Jinan. Jinan karstic spring region is divided into three major hydrogeological types: the alluvial plain of Yellow River (Region I), the north boundary of the Mount Tai (Region II), and the piedmont alluvial plain (Region III) [31,32]. These three regions account for 30%, 50% and 20% of the total area of Jinan. Each of the major regions could be further partitioned into several sub-regions based on topography, lithology, and geological structure, as shown in Figure 4. The specific division of the spring region is summarized in Table 1. The ten main spring groups are completely distributed to the south of the Yellow River, most of which are centered on the urban area of Jinan, in sub-Region II3. Sub-Region II3 is well-known as the typical geological region of karstic spring basin, where the springs of Jinan mainly discharge. Figure 5 shows the water level of Sprouting Spring in 2016. JG1, JG2, and JG3 are the three monitoring points. As shown in this figure, there are similar trends for JG1 and JG3. It also reveals that the water level of Sprouting Spring is highest during August and September.

**Figure 6:**
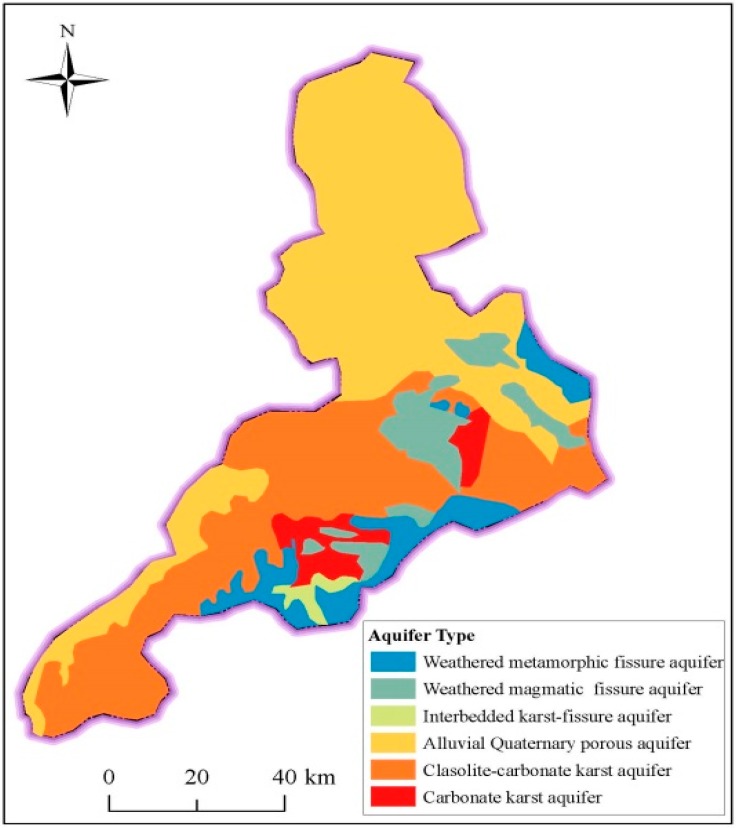
Spatial distribution of aquifer types in Jinan.

Figure 6 shows the spatial distribution of aquifer in Jinan. The stratigraphic units can be grouped into six categories of aquifer based on the general hydraulic characteristics. The karstic aquifers formed by the carbonates of the Cambrian and Ordovician can store and transmit large quantities of groundwater. Karstic fissures are interconnected within the piedmont of Mount Tai and then formed in the south–north direction channels for the discharge of karstic water. On the influence of the monoclinal structure, the karstic aquifers incline downward into the Quaternary deposit in the north part of the basin, which provides the head pressure to induce the spewing of spring groups. On the north bank of Yellow River, the thick and widespread distributed alluvial silt and sand provide barriers to further movement of karstic groundwater into the northern plain, where only phreatic water is stored in the Quaternary porous aquifer. Pumping from these aquifers may significantly impact surroundings [36,37,38]. In some rocks of ancient geological age, groundwater mainly occurs in the magmatic fissured aquifers of the piedmont alluvial plain and in fissures of metamorphic rocks of Mount Tai [31,32].

#### 2.2.3. Engineering Geology 

**Figure 7:**
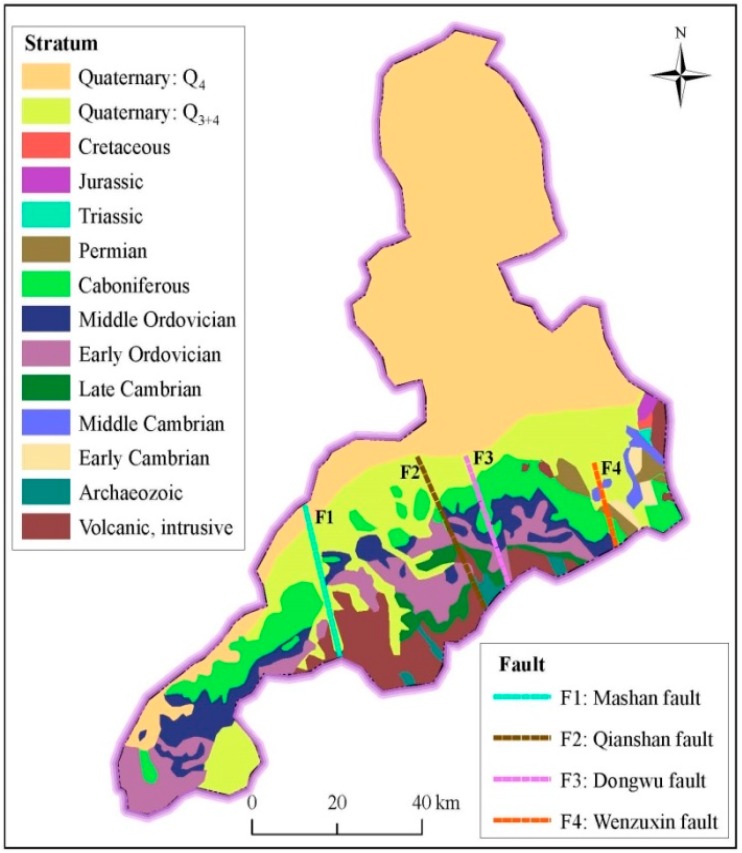
Spatial distribution of strata in Jinan.

Regional geological condition is a critical factor, which will affect the underground constructions and the long-term behavior of underground metro system. Figure 7 depicts the spatial distribution of geological strata of Jinan. The stratum outcrops from newest to oldest, and north to south, include: Tertiary system and Quaternary of Cenozoic; Triassic System, Jurassic System and Cretaceous System of Mesozoic; Cambrian System, Ordovician System, Carboniferous System, and Permian System of Paleozoic; and Taishan group of Archaeozoic [1,31,32]. Several large-scale north-northwest (NNW) distributed active faults have developed. These faults are parallel distributed, from west to east, separating the whole monoclinal structural zone into four fault blocks. As shown in Figure 3, the four active faults are F1 (Mashan Fault), F2 (Qianshan Fault), F3 (Dongwu Fault), and F4 (Wenzuxin Fault). The current geology and landform in the northern areas are mainly shaped by long-term tectonic movement, especially by magmatic intrusions in Yanshan movement during Cretaceous period. The rock mass formed by intrusion is widespread over the piedmont of Mount Tai. In the vast areas of Regions II and III, the upper strata are mainly alluvial sand and silt deposited during the Quaternary period, as brought by the washing action of Yellow River. These silt deposit has time dependent behavior, which may become internal cause long-term settlement of underground tunnels [39,40,41,42,43,44].

#### 2.2.4. Distribution of Metro Lines

**Figure 8:**
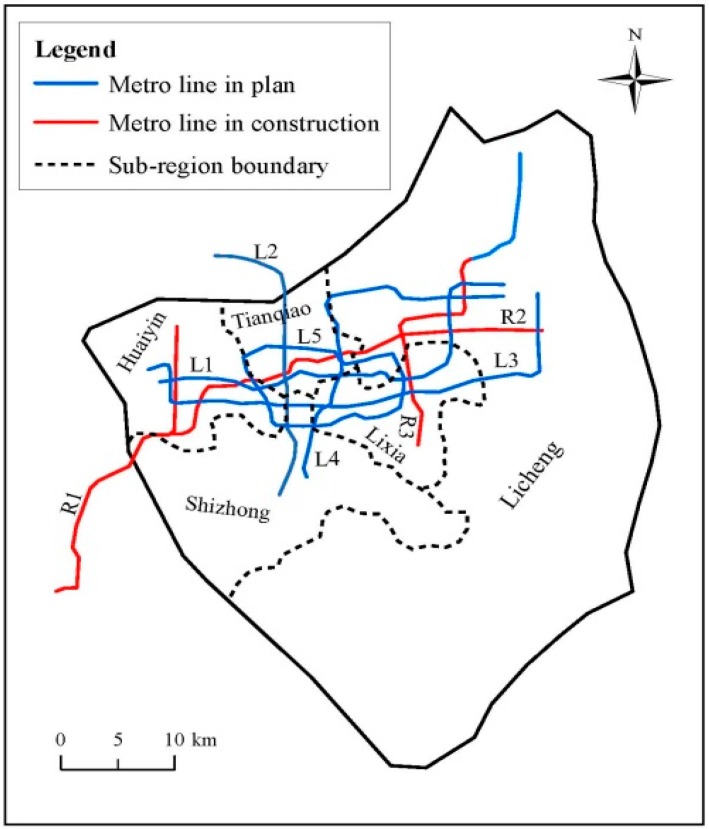
Spatial distribution of metro line in the urban area of Jinan.

With progress of urbanization, several metro tunnels have been constructed or are under construction in Jinan City to alleviate traffic tension. Figure 8 depicts the distribution of metro lines in Jinan. The main concentration of metro lines in Jinan is in urban area, where the four major spring groups’ outcrops locate, as mentioned before. Interaction between the construction of metro lines and the characteristics of topography, hydrology, and geology results in the specific effects of metro system on the local groundwater system, e.g., blocking groundwater seepage, depletion of springs, etc. 

## 3. Methodology

### 3.1. Risk Assessment Model

**Figure 9:**
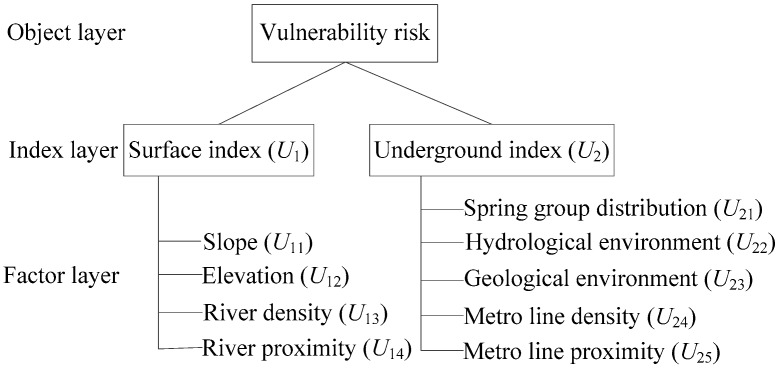
Structure of assessment index system.

The objective for risk assessment is to protect the natural environment of spring discharge routine underground. Regional disaster risk assessment system is represented by the vulnerability of disaster-bearing body, which can be characterized as a system including surface index, underground index, and anthropic activities. To assess the regional vulnerability, the surface and underground indices are used to construct a comprehensive assessment index system. Therefore, the object layer is the risk of vulnerability in the assessment index system, which was constructed by surface index and underground index. The structure of the assessment index system is shown in Figure 9. To reflect the influence of metro system, the metro system is considered as a factor in underground index in the assessment system. The assessment model can be expressed as follows:

(1) $$R=f(v)=\sum_{i=1}^{i=n}S_{i}\times s_{i}+\sum_{i=1}^{i=n}U_{i}\times u_{i}$$

where *R* is the risk level, which is expressed by the function of *f(v)*; *v* represents the vulnerability of bearing body; *S_i_* and *U_i_* are the surface index and underground index, respectively; *s_i_*, and *u_i_*, are the corresponding weight coefficient of each index; and *n* is the number of index.

### 3.2. Processing of Each Index

#### 3.2.1. Surface Index

**Figure 10:**
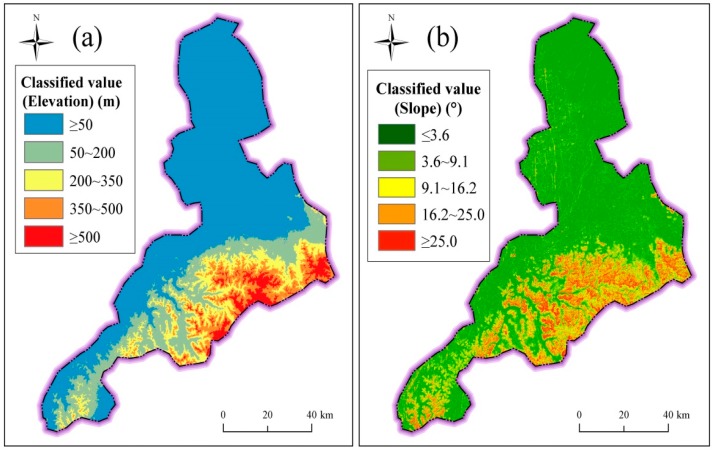
Classification of topography: (**a**) elevation; and (**b**) slope.

Topography has significant influence on the development of disaster, e.g., flooding hazards, debris flows, and landslides. The characteristics of elevation and slope are widely used to reflect the topography. To guarantee the accuracy of assessment result, the elevation and slope are divided into five levels with the resolution of 100 m. Figure 10 shows the classification of elevation and slope. Surface with a low elevation and flat slope has a high risk of disasters. The region of Jinan City has an obvious difference of elevation and slope from north to south. 

**Figure 11:**
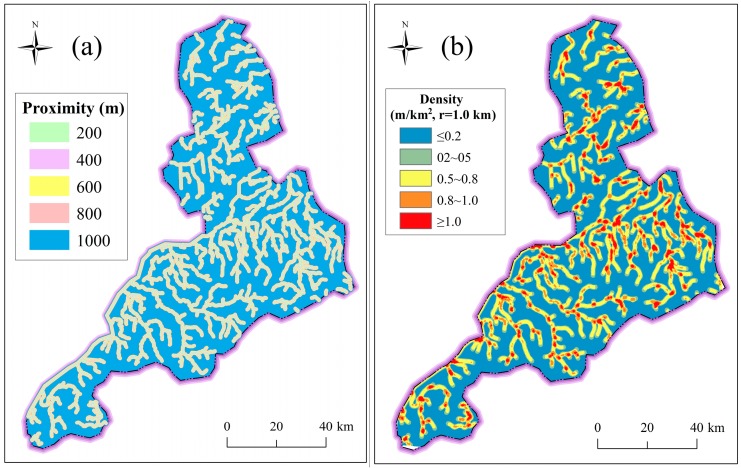
Characteristics of river system: (**a**) river proximity; and (**b**) river density.

In addition to elevation and slope, the river system also has direct effects on the forming of disasters, especially flooding hazards. The river system can drain rainwater during rainstorms, but can also induce flooding when the tide rises over its embankment top. The river proximity and river density are used to consider the influence of river system from individual river channel and multiple river channels. Figure 11 shows the characteristics of river system. River proximity represents the distance to the closet river channels; it was extracted using the Multiple Buffer Operator with distances of 200 m, 400 m, 600 m, 800 m, and 100 m. River density indicates the length of river channels per unit area; it was extracted by the Line Density Function using a radius of 1.0 km.

#### 3.2.2. Underground Index

**Figure 12:**
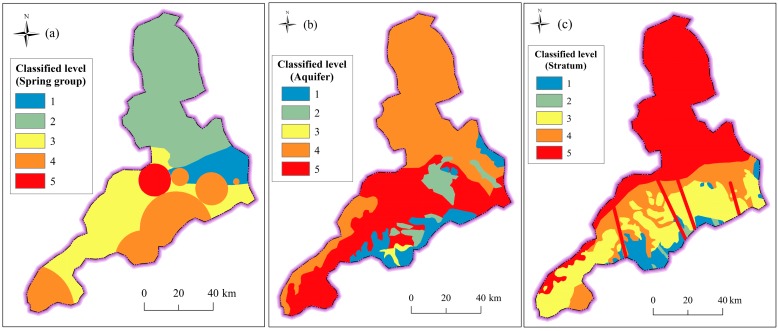
Classified level of underground index: (**a**) spring groups; (**b**) aquifer; and (**c**) stratum.

Underground index includes the condition of hydrogeology and geology. Table 2 tabulates the water balance of groundwater in Jinan. According to the water balance of groundwater system, three types of hydrogeological partition, i.e., Yellow River (I), Mount Tai (II), and the piedmont alluvial plain (III), are assessed as level 2 (Low), level 3 (Medium) and level 1 (Very low), respectively. Since there are significant effects of spring groups, the four main spring groups are assessed as level 5 (very high), and other spring groups are assessed as level 4 (High). Figure 12a shows the classified level of spring groups in Jinan City. According to the discharge capacity of aquifer, the risk level of aquifer was classified into five levels. Table 3 lists the characteristics and classification of aquifer in Jinan City. Figure 12b shows the spatial distribution of classified aquifer in Jinan. The karstic aquifer with a high discharge capacity is assessed at level 5, while the fissure water with a low discharge capacity is assessed at level 1. According to geological era of outcrop stratum from newest to oldest, the risk level ranged from level 1 to level 5. Figure 12c depicts the spatial distribution of classified stratum. In addition, regions where faults develop are often vulnerable to disasters. To reflect the influence of faults, the Multiple Buffer Operator with the distance of 1 km was used to calculate the affected areas, which were assessed at level 5.

**Figure 13:**
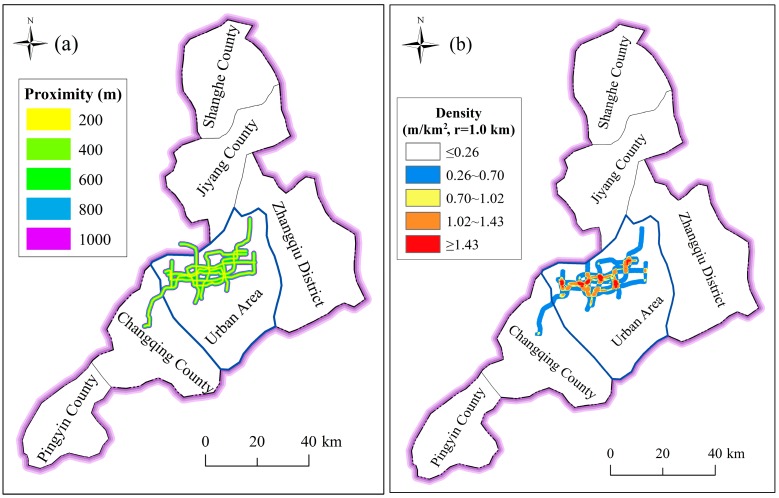
Spatial distribution of metro line proximity and density: (**a**) proximity; and (**b**) density.

In addition to the condition of hydrogeology and geology, the metro system, which represents anthropic activities, is also regarded as an underground factor. To assess and predict the risk of metro line construction, this paper considered both metro lines under construction and the planned metro lines. According to previous research on the long-term settlement of metro lines, the impact area of metro lines can reach 1000 m [4,8]. Figure 13 shows the spatial distribution of metro line proximity and density. To consider the affected range of metro line, the values of proximity of metro lines are set as 200 m, 400 m, 600 m, 800 m, and 1000 m, respectively. The density of metro lines was computed using a radius of 1 km. 

### 3.3. Weight Calculation by AHP

To assess the risk and impacts of metro line construction on the groundwater system, Analytic Hierarchy Process (AHP) is used to calculate the weight coefficient of each factor. The main steps of AHP are as follows: (a) establish the hierarchical structure according to the assessment index system; (b) create the judgment matrix as in Equation (2); (c) calculate the weight coefficient of each factor using Equation (3); (d) perform the consistency test of judgment matrix as in Equation (4); and (e) calculate the combination weighting of factors at all levels as well as the total consistency test [45,46]. The judgment matrixes are listed in Table 4 and Table 5. As tabulated in Table 4 and Table 5, the values of CR for surface index and underground index are less than 0.1, which means that the judgment matrix is consistent. The weights determined using this method are listed in Table 6.

(2) $$A_{p}=(a_{ij})={\left(\begin{array}{cccc} a_{11} & a_{12} & \cdots & a_{1n}\\ a_{21} & a_{22} & \cdots & a_{2n}\\ \vdots & \vdots & \ddots & \vdots \\ a_{m1} & a_{m2} & \cdots & a_{mn} \end{array}\right)}_{p}$$

where *A_p_* is the judgment matrix, and *a_ij_* is the element of judgment matrix, which is composed by each factor of assessment index.

(3) $$f_{i}=\frac{M_{i}}{\sum_{i=1}^{n}M_{i}}=\frac{\sqrt[{n}]{\prod_{j=1}^{n}a_{ij}}}{\sum_{i=1}^{n}M_{i}}$$

where *M_i_* is the *n*^th^ root of each product line element.

(4) $$CR=\frac{CI}{RI}$$

where $CI=(\lambda_{\max}-n)/(n-1)$, and *λ*_max_ is the largest eigenvalue of the judgment matrix; which can be calculated based on Equation (5). *RI* is the average random consistency index.

(5) $$\lambda_{\max}=\sum_{i=1}^{n}\frac{\sum_{j=1}^{n}a_{ij}w_{i}}{nw_{i}}$$

### 3.4. Normalization

**Figure 14:**
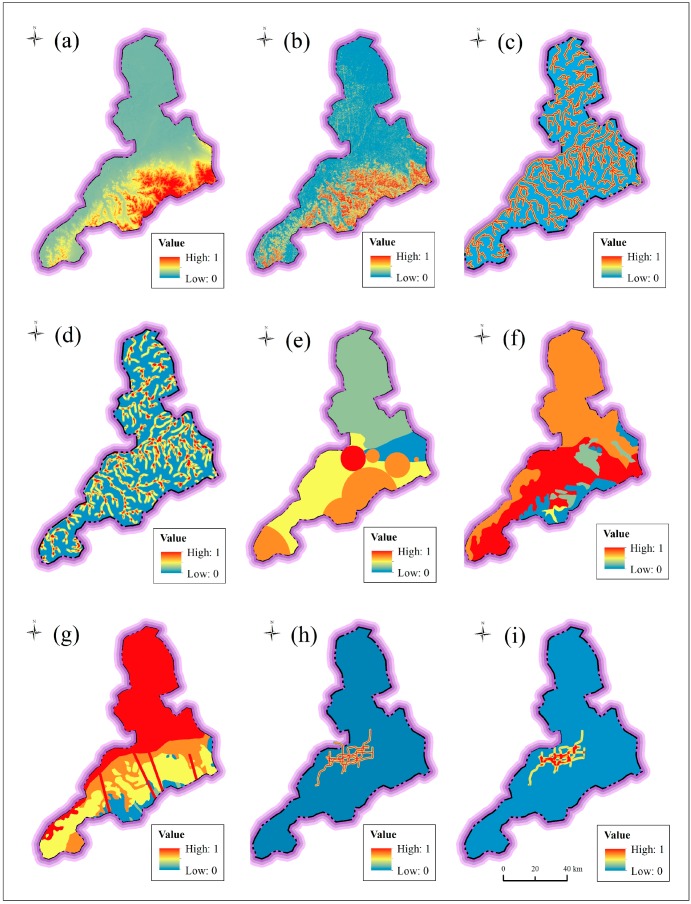
Normalized factors of assessment system: (**a**) elevation; (**b**) slope; (**c**) river proximity; (**d**) river density; (**e**) spring groups; (**f**) hydrogeology; (**g**) geology; (**h**) metro line proximity; and (**i**) metro line density.

To facilitate the comparability of different factors, each factor was normalized over the range from 0 to 1. In the index system of assessment, disaster risk will decrease with the increase of topographic elevation and slope, river density, and metro line density. These four factors are negative factors. The other factors are positive factors. The positive factors and negative factors are normalized using Equations (6) and (7), respectively. The final normalized factors are shown in Figure 14.

(6) $$i_{ij}=\frac{a_{ij}-a_{p\min}}{a_{p\max}-a_{p\min}}$$

(7) $$i_{ij}=\frac{a_{p\max}-a_{ij}}{a_{p\max}-a_{p\min}}$$

where *i_ij_* is the normalized value of the index matrix; *a_ij_* is the original value of index; *a*_*p*max_ is the maximum value of the judgment matrix of index *p*; and *a*_*p*min_ is the minimum value of the judgment matrix of index *p*.

## 4. Results

### 4.1. Assessment of Surface Index

**Figure 15:**
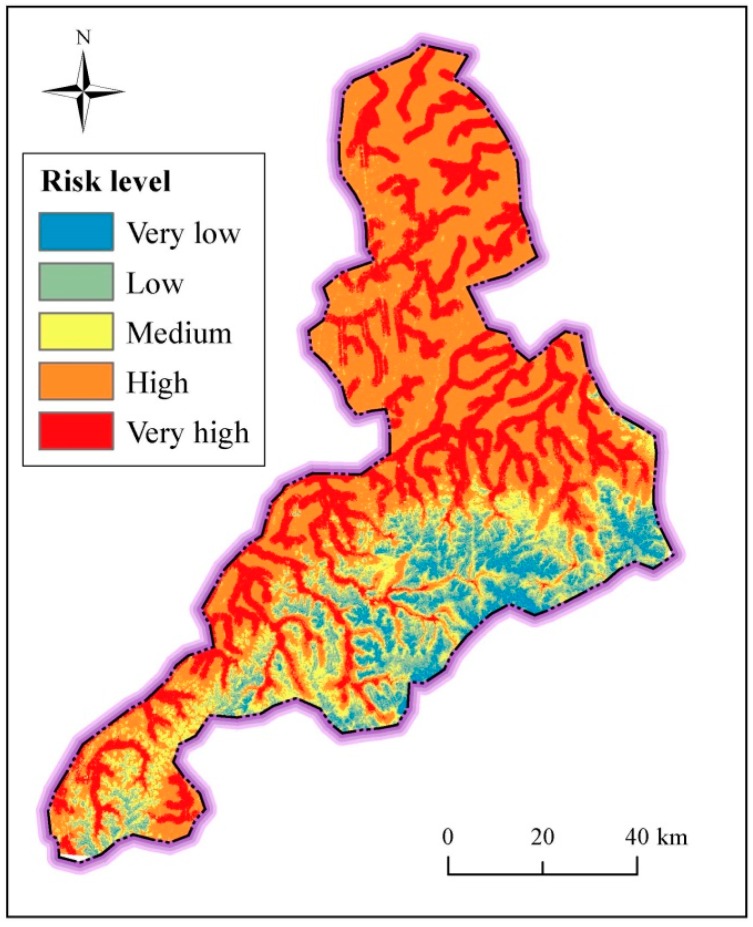
Spatial distribution of risk level of surface index in Jinan.

In the assessment system, the surface index includes the topographical elevation and slope, river proximity and density. Based on the weight coefficient and grid cell data of normalized factors, the spatial distribution the risk level of surface index is obtained (Figure 15). Figure 15 shows the spatial distribution of the risk level of surface index in Jinan. As shown in Figure 15, the risk level of surface index decreases from north to south. High-risk level is mainly located in the north and southwest along Yellow River, where there is a high density of river system and a low elevation. There is an obvious transition trend from high risk level to low risk level in the south. Areas in a low risk level distributes in the high elevation. Table 6 tabulates the statistical risk level of surface index in Jinan. As listed in Table 7, the high and very high risk level is over 98% in Shanghe County and Jiyang County, where there are flat topography and intensive river system. The areas with the high and very high risk level exceed 66% in Zhangqiu District and Pingyin County, where there is the transition region between flat plain and mountain area. The regions with the high and very high risk level exceed 51% in urban area and Changqing County, where the elevation is relatively low. According to these results, the region with a flat topography and adjacent with river system always has a high risk of disaster. 

### 4.2. Assessment of Underground Index

**Figure 16:**
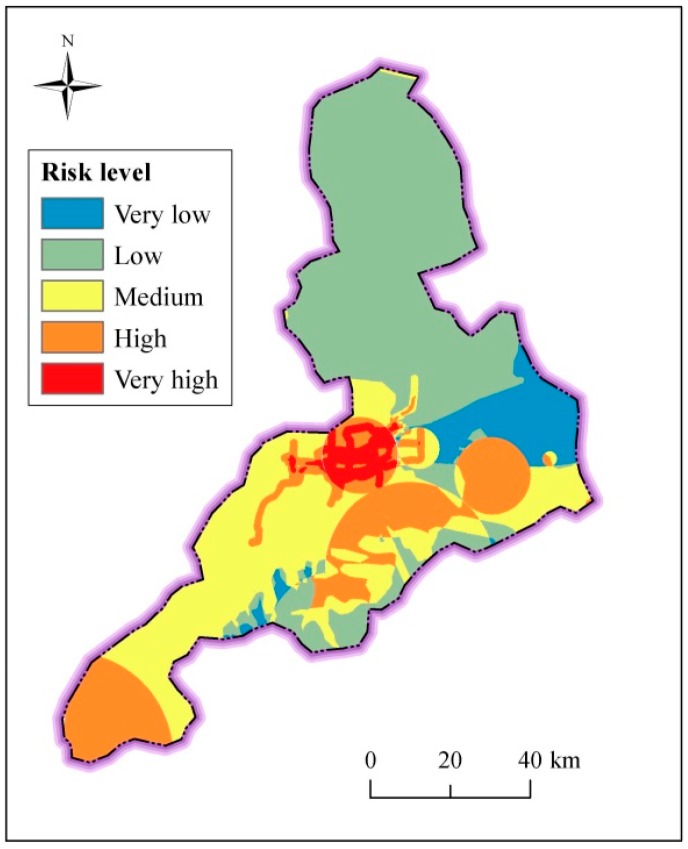
Spatial distribution of risk level of underground index.

Underground index includes spatial distribution of spring groups, characteristics of hydrogeology and geology, and metro line proximity and density [47,48]. Figure 16 shows the assessed result of risk level of underground index. As shown in Figure 16, the region with the four main spring groups has high risk level of underground index, while some parts of the piedmont alluvial plain (III) have the low risk level. In addition, some parts with distribution of metro line also have high risk level. The north region with Quaternary stratum has relatively low risk level. The north region, where there are spring groups and active faults have relative high risk level. Overall, the central area, where metro lines are being constructed, has high risk level of underground index. Table 8 tabulates statistical risk level of underground index in Jinan City. As listed in this table, the areas with high-risk level mostly locate in the south part of Jinan. The regions of high and very high risk level exceed 56% in urban area and Pingyin County, where many spring groups and active faults distribute. The area with low-risk level mostly locates in the north flat plain of Jinan. Therefore, the areas with the distribution of spring groups and active faults are often vulnerable to bear the disturbance of anthropic activities.

### 4.3. Spatial Distribution of Risk Assessment

**Figure 17:**
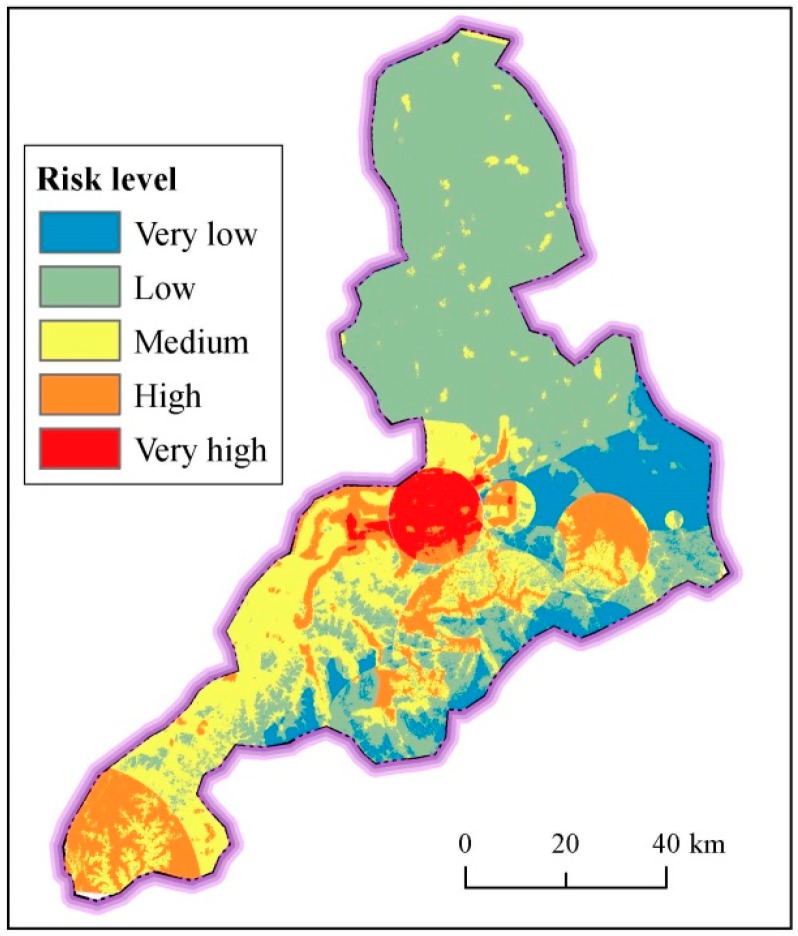
Spatial distribution of risk level in Jinan City.

The final risk level can be evaluated based on the previous assessment results. Figure 16 shows the spatial distribution of risk level in Jinan. As shown in Figure 17, the area with the very high risk level is concentrated in central Jinan, where there is the densest distribution of metro lines. The regions with the second most high risk level areas are mostly distributed where there are spring groups and active faults. The areas subject to medium risk level are mainly located in the south and southwest along with Yellow River. The very low risk level area is located in the region of piedmont alluvial plain (III). The second most low risk level areas are located in the north of Jinan City with the Quaternary stratum.

Table 9 tabulates the comprehensive statistical risk level in different districts. In terms of administrative division (Table 5), the regions with very high and high risk level exceed 33%, which belongs to urban area. Pingyin County has 42.72% high risk level coverage, and is in the southwest of Jinan City. Because of the amount of spring groups and four active faults in Pingyin County and urban area, the vulnerability of underground activities (e.g., metro line construction) in these areas is obvious. Therefore, the comprehensive risk level is high. The regions with medium risk are mostly located in the Changqing County, Pingyin County and urban area. The low risk regions are located in Shanghe County, Jiyang County and Zhangqiu County. Overall, the risk level is high in the south region where the condition of hydrogeology and geology are vulnerable, while low in the north plain region with low elevation and Quaternary stratum, and the very high risk is located in the transition zone between the mountains and plains.

### 4.4. Risk Level of Metro Line

**Figure 18:**
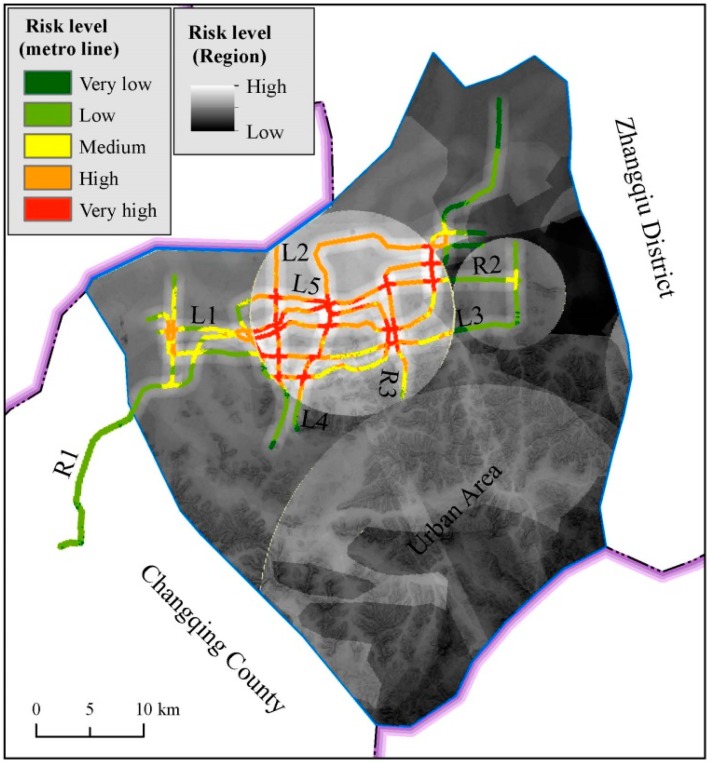
Risk level of metro line in urban area.

Disaster risk assessment results can identify safe or unsafe regions of urban area during development. Therefore, the risk distribution map provides meritorious information for land use planning and targeting areas for prioritizing risk reduction measures. According to the assessment result of risk level in the whole Jinan City, the urban area, where the metro line is under construction, has high risk. Figure 18 shows the risk level in urban area. As shown in Figure 18, four main spring groups are located in the high risk region. Within this high risk region, metro lines R1, R2, and R3 (under construction) and metro lines L1, L2, L3, L4 and L5 (planned) have very high and high risk. These results suggest that risk control measures should be intensively adopted for metro line construction within this area to mitigate disaster losses. The other parts of the metro lines have relatively low risk. As the key underground infrastructure, metro line construction is developing rapidly. Jinan City has abundant groundwater and spring groups, which have significant influence on metro line construction. Therefore, it is indispensable to assess the risk and impacts of metro line construction to groundwater system in Jinan.

## 5. Discussion

### 5.1. Uncertainties of AHP

There are many uncertainties in the disaster risk assessment process. The present assessment model may not fully express the true situation since a real disaster system is complicated and fuzzy [49]. This assessment model only includes nine factors associated with disaster risk in metro line construction. In addition, the weight coefficient of each factor during assessment is partly based on expert experience and documents investigation, which lack precise calculation. Although these assessment results are uncertain, the spatial distribution of risk level still has significant suggestions to the urban development, especially for the construction of metro lines.

### 5.2. Suggestive Countermeasures

**Figure 19:**
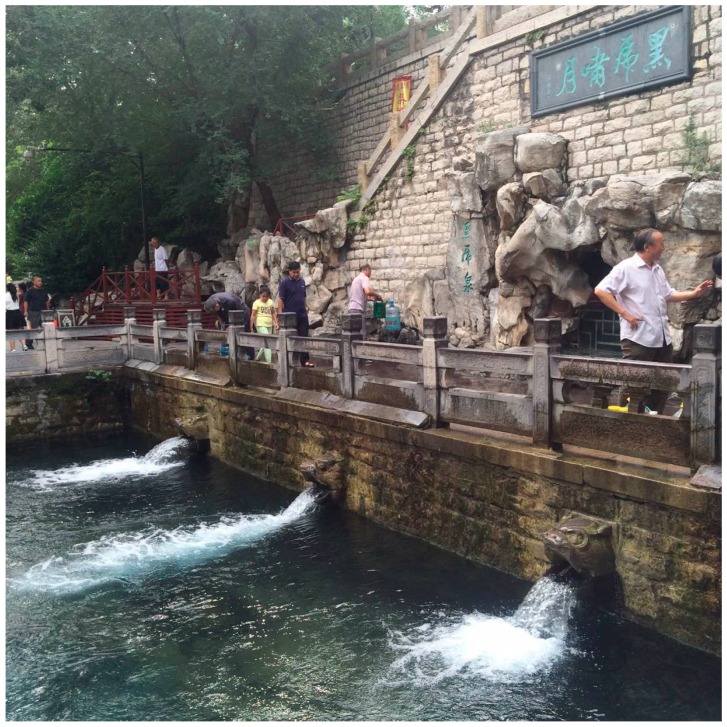
Heihu Spring in Jinan City.

Metro line construction causes disturbance to adjacent underground environment, and also has a further influence on local geoenvironment. In the assessment system, the weight coefficient of metro line is 0.21, which is 13% of all indices. The result shows that the effects of metro line construction to geoenvironment are up to 13%. Therefore, it is crucial to protect geoenvironment (e.g., spring groups) during the process of metro line construction. There are several suggestions to protect spring groups: (a) avoid dewatering during excavation or tunneling; (b) avoid blasting construction in the adjacent areas where spring groups distribute; (c) use sustainable materials instead of grouting materials to avoid influencing groundwater quality in the spring groups region; and (d) construct ground lines instead of underground lines by viaduct to protect groundwater system in the densest distribution of spring groups [50,51,52,53,54]. When the underground lines are unavoidable, the following innovative technical measures are proposed for this special geology and hydrogeology in Jinan: (1) setup of pumping recharge system; and (2) construct by-pass of groundwater for underground tunnel constructed aquifer. Two new features of the pumping recharge system are: (i) water treatment facility can improve the water quality; and (ii) pressure control box can control the volume into the recharge well intelligently. There are two situations for construction of groundwater pathway: (a) groundwater pathway in a circular tunnel via embedding still pipe; and (b) groundwater pathway in a waterproof curtain of metro station. The groundwater pathway allows the groundwater to pass through the barriers. Thus, the barrier effect of the metro line is reduced; thus, groundwater can freely seep through the constructed underground structures. After the aforementioned measures were conducted, the spring groups were maintained well. Figure 19 shows the spouting of Heihu Spring. 

## 6. Conclusions

This study used the method of AHP and GIS technology to assess the environmental impact risk level of metro line construction on groundwater system. From the perspective of surface index and underground index, nine factors were considered in the risk assessment model: topographical elevation and slope, river proximity and density (surface index), characteristics of spring groups, hydrogeology, geology, and metro line proximity and density (underground index). The major conclusions can be drawn as follows:

1. The AHP method incorporated into GIS can make a comprehensive and reasonable assessment result of risk level of groundwater seepage environment in Jinan City. The method can also be applied to other region based on different characteristics of environment. The results from the proposed method are useful for practical decision-making during urban planning.
2. The assessment of surface index shows that the regions with the high and very high risk level exceed 98% in Shanghe County and Jiyang County, where there are flat topography and intensive river system. The assessment of underground index shows that the areas with the high and very high risk level exceed 56% in urban area and Pingyin County, where distribute many spring groups and active faults. The areas with the distribution of spring groups and active faults are often vulnerable to bear the disturbance of anthropic activities.
3. The comprehensive assessment result shows that about 14% of the urban area belongs to very high risk level, where the main four spring groups distributed. The regions of relatively high risk are 20% in urban area, 9.46% in Changqing County and 43% in Pingyin County, where the characteristics of hydrogeology and geology are vulnerable for metro line construction.
4. According to the assessment result of risk level in the whole Jinan City, in the high risk region where there are four main spring groups; metro lines R1, R2, and R3 are under construction; and metro lines L1, L2, L3, L4 and L5 are planned have very high and high risk. It is suggested to construct elevated tunnel rather than underground tunnel to protect spring groups.

## Figures and Tables

**Table 1:** Table of hydrogeological partition of Jinan karstic spring region.

Code	Region Name	Type	Code	Sub-Region
I	Alluvial plain of Yellow River	Pore water	I1	Shang River area
			I2	Jiyang River area
			I3	North area of Xiaoqing River
II	North boundary of the Mount Tai	Karstic water	II1	Pingying monoclinic area
			II2	Changqing monoclinic area
			II3	Jinan monoclinic area
			II4	Guodian monoclinic area
			II5	Monoclinic area around Ming River
			II6	Monoclinic area around Xiaoqing River
III	Piedmont alluvial plain	Pore water	III	Piedmont alluvial plain

**Table 2:** Water balance of groundwater in Jinan karstic spring basin.

Region	Sub-Region	Recharge	Discharge	Exploitable Capacity	Current Exploitation	Risk Level
I	I1	69.77	28.81	40.96	32.05	2
	I2	73.46	24.83	48.63	20.82	
	I3	23.23	9.56	13.67	4.46	
	Subtotal	166.46	63.20	103.26	57.33	
II	II1	13.92	1.15	12.77	7.09	3
	II2	15.91	-	15.91	4.93	
	II3	58.99	23.97	35.02	36.27	
	II4	34.55	8.01	26.54	32.21	
	II5	32.05	17.39	14.66	14.48	
	II6	30.85	9.06	21.79	13.64	
	Subtotal	186.27	59.58	126.69	108.62	
III	III	48.22	16.49	31.73	40.33	1

Note: The amount of water in this table is counted as million cubic meters per day (10,000 m^3^/day).

**Table 3:** Hydraulic characteristics and classification of aquifer in Jinan City.

Aquifer Type	Geological Time	Discharge Capacity (m3/day)	Class
Fissure water in metamorphic rocks and magmatic rocks	σ, Ar	Generally: <100	1
Fissure water in Weathered magmatic	Є1, Є2, Є3	Generally: <100	2
		Partially: 100–500	
Interbedded karst-fissure aquifer	Є2z, Є3f	Generally: <500	3
		Partially: 500–1000	
Alluvial Quaternary porous aquifer	Q4	Generally: <200	4
	Q2	Piedmont: 50–300	
	Q3	Alluvial fan: 1000–2000	
Karstic aquifer in carbonate	Є2z, Є3f, O1, O2	Hills: 100–500	5
		Piedmont: 1000–5000	
		Partially: >10,000	

**Table 4:** Judgment matrix and validation of consistence for surface index.

	U_11_	U_12_	U_13_	U_14_	*w_i_*
U_11_	1	2	3	3	0.475
U_12_	1/2	1	1	2	0.231
U_13_	1/3	1/3	1	4	0.188
U_14_	1/3	1/2	1/4	1	0.105

*CR* = 0.0101 < 0.1.

**Table 5:** Judgment matrix and validation of consistence for underground index.

	U_21_	U_22_	U_23_	U_24_	U_25_	*w_i_*
U_21_	1	1	2	3	3	0.321
U_22_	1	1	1	2	3	0.259
U_23_	1/2	1	1	2	3	0.208
U_24_	1/3	1/2	1/2	1	1	0.109
U_25_	1/3	1/3	1/2	1	1	0.101

*CR* = 0.0789 < 0.1.

**Table 6:** Index system for vulnerability assessment in Jinan.

Object Layer	Sub-Object Layer	Factor Layer	*w_i_*	*w_i_*
Geoenvironment risk	Surface index U_1_	Topographical slope (U_11_)	0.475	0.363
		Topographical elevation (U_12_)	0.231	
		River density (U_13_)	0.188	
		River proximity (U_14_)	0.105	
	Underground index U_2_	Spring group distribution (U_21_)	0.321	0.637
		Hydrogeology (U_22_)	0.259	
		Geological environment (U_23_)	0.208	
		Metro line density (U_24_)	0.109	
		Metro line proximity (U_25_)	0.101	

**Table 7:** Statistical risk level of surface index in different district.

District	Risk Coverage of Surface Index (%)				
	Very Low	Low	Medium	High	Very High
Shanghe County	0	0.01	0.58	65.09	34.32
Jiyang County	0	0.02	10.02	66.54	32.42
Zhangqiu District	7.79	13.12	12.53	40.63	25.93
Urban Area	10.90	19.58	17.42	28.83	23.27
Changqing County	5.03	16.15	21.72	33.06	24.04
Pingyin County	0.1	8.13	23.64	42.62	24.65

**Table 8:** Statistical risk level of underground index in different district.

District	Risk Coverage of Underground Index (%)				
	Very Low	Low	Medium	High	Very High
Shanghe County	99.18	0.02	0	0	0
Jiyang County	73.97	26.03	0	0	0
Zhangqiu District	67.77	15.14	17.09	0	0
Urban Area	0.9	8.43	27.55	36.84	26.28
Changqing County	0.02	24.41	65.54	10.03	0
Pingyin County	0.1	0.02	43.22	56.66	0

**Table 9:** Comprehensive statistical risk level in different district.

District	Comprehensive Risk Coverage (%)				
	Very Low	Low	Medium	High	Very High
Shanghe County	0.23	96.48	3.28	0.01	0
Jiyang County	0.16	94.56	5.27	0.01	0
Zhangqiu District	31.24	49.70	8.71	10.35	0
Urban Area	9.23	25.34	31.50	20.04	13.89
Changqing County	10.54	29.11	50.90	9.46	0
Pingyin County	0	7.31	49.97	42.72	0
